# Metagenomic next-generation sequencing identifies native valve Aspergillus fumigatus endocarditis with cerebral involvement: a case report

**DOI:** 10.3389/fcvm.2025.1487543

**Published:** 2025-03-04

**Authors:** Weibing Wu, Jingjing Xu, Jianling Ruan, Baoping Tian, Nanxia Xuan

**Affiliations:** ^1^ Department of Critical Care Medicine, Qingyuan People’s Hospital, Lishui, China; ^2^ Department of Radiology, The Second Affiliated Hospital of Zhejiang University School of Medicine, Hangzhou, China; ^3^ Department of Ultrasound in Medicine, Qingyuan People’s Hospital, Lishui, China; ^4^ Department of Critical Care Medicine, The Second Affiliated Hospital of Zhejiang University School of Medicine, Hangzhou, China

**Keywords:** mNGS, case, aspergillus endocarditis, intracranial infection, diagnosis

## Abstract

Aspergillus endocarditis is a rare but highly fatal condition, particularly in immunocompromised patients. This case report describes a 74-year-old male with native valve Aspergillus fumigatus endocarditis and intracranial infection. Diagnosis was complicated by atypical presentation and negative blood cultures, but metagenomic next-generation sequencing (mNGS) enabled rapid identification of the pathogen. This case is notable for being the first to document Aspergillus fumigatus endocarditis with cerebral involvement confirmed by mNGS, highlighting the importance of early diagnosis and advanced diagnostic tools in improving outcomes.

## Background

Fungal endocarditis has a low incidence and carries a mortality rate exceeding 50% (1). It primarily affects patients with compromised immunity (2). The most common pathogen in fungal endocarditis is Candida, followed by Aspergillus and Histoplasma (3, 4). Aspergillus endocarditis often occurs in patients with prosthetic valve implants or intravascular devices, as well as those who have undergone solid organ transplantation (4, 5). However, primary Aspergillus endocarditis of native valves is rarely reported. The clinical presentation of Aspergillus endocarditis is atypical, and blood cultures are often negative, making early etiological diagnosis difficult and leading to treatment delays (1, 3). Herein, we report a case of Aspergillus fumigatus endocarditis with intracranial infection in a native valve from a mountainous region in China. We were able to rapidly identify the pathogen and concomitant infection sites through metagenomic next-generation sequencing (mNGS) for precise treatment. To our knowledge, this is the first reported case of Aspergillus fumigatus endocarditis with intracranial infection in a native valve confirmed by mNGS. We obtained written informed consent from the patient's authorized representative (as the patient was unable to provide consent personally) for the publication of this case report.

## Presentation of case

A 74-year-old male was admitted with chest tightness, dizziness, and fatigue for 5 days. He had a history of prostate cancer, treated with bicalutamide and goserelin. Six months ago, the patient contracted COVID-19. After treatment, the viral nucleic acid test turned negative. He had no prior cardiac issues, implants, or surgeries and lived in a mountainous area where edible fungi are grown.

**Figure 1:**
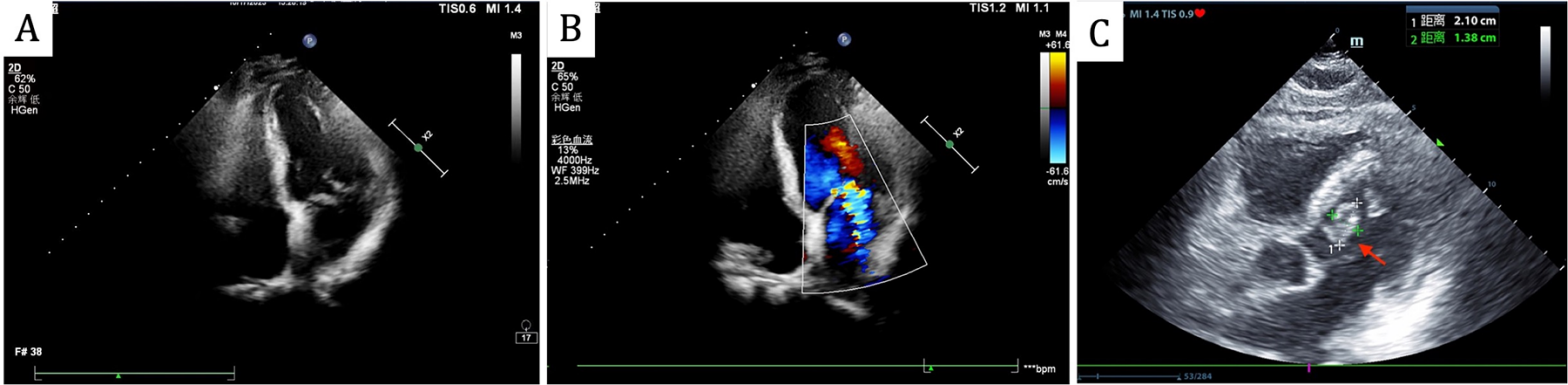
Echocardiography revealed partial “crab-like” movement of the mitral valve's anterior leaflet, and mild to moderate regurgitation on admission **(A,B)**, the follow-up echocardiogram revealed a mitral valve mass, as indicated by the red arrow **(C)**.

On admission, he had a fever (37.9℃) and a systolic murmur. Lung examination showed coarse breath sounds. Laboratory results indicated elevated white blood cells (11.6 × 10^9^/L), neutrophils (80.9%), C-reactive Protein (11.9 mg/L), Erythrocyte Sedimentation Rate (40 mm/h), and B-type Natriuretic Peptide (674 pg/ml). Echocardiography revealed an Ejection Fraction of 61%, partial “crab-like” movement of the mitral valve's anterior leaflet, and mild to moderate regurgitation (Figures 1A,B). Chest Computed Tomography (CT) showed chronic bronchitis.

**Figure 2:**
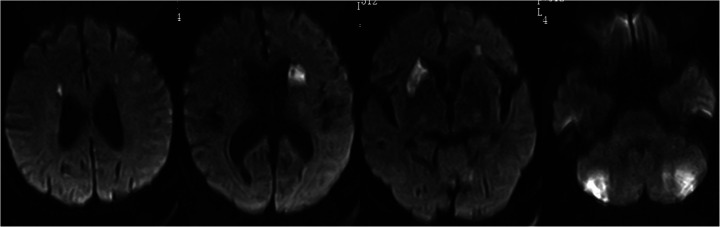
MRI DWI shows multiple acute infarctions in the bilateral periventricular regions, basal ganglia, and cerebellum, with a few lacunar infarctions in the bilateral frontal and parietal lobes.

Despite cefoperazone-sulbactam therapy, the patient continued to have fever and rising inflammatory markers. He experienced intermittent limb weakness and delirium, resolved spontaneously after lasting for several minutes. On the 7th day, Magnetic Resonance Imaging Diffusion-Weighted Imaging (MRI DWI) revealed multiple acute cerebral infarctions and lacunar infarcts (Figure 2). Aspirin and clopidogrel were added, but he later developed coma and respiratory failure, necessitating intubation and Intensive Care Unit admission.

**Figure 3:**
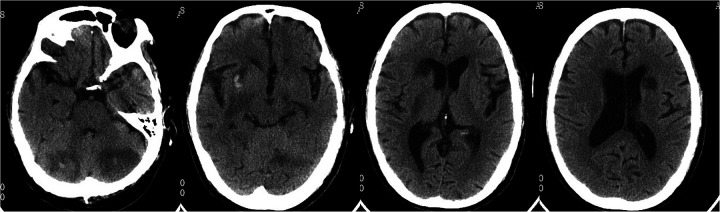
Follow-up head CT revealed multiple cerebral infarctions in the bilateral periventricular regions, basal ganglia, and cerebellum, with a small amount of bleeding in the right basal ganglia infarction area.

Head CT showed new hemorrhage in the right basal ganglia (Figure 3). Lumbar puncture and mNGS of Cerebrospinal Fluid (CSF), peripheral blood, and bronchoalveolar lavage fluid were performed. The mNGS results were as follows: 434 sequences of Aspergillus fumigatus in cerebrospinal fluid, 12 sequences of Aspergillus fumigatus in peripheral blood, and no Aspergillus fumigatus detected in bronchoalveolar lavage fluid (Table 1). Subsequent to the mNGS finding, a follow-up echocardiogram was done which revealed a mass on the mitral valve of size 1.38 cm × 2.10 cm suggestive of a vegetation (Figure 1C). Next, voriconazole was initiated to treat suspected Aspergillus endocarditis with embolization and meningoencephalitis. The patient remained comatose, and on the 14th day, with bilateral pupillary dilation and a Glasgow Coma Scale score of 3, the family opted for palliative care. CSF and blood bacterial cultures were negative, and the serum G + GM test was also negative. The G test, also known as the 1,3-β-D-glucan test, detects the 1,3-β-D-glucan component in fungal cell walls. After phagocytes engulf fungi, they continuously release this substance, increasing its concentration in blood and bodily fluids. 1,3-β-D-glucan specifically activates the G factor in Limulus amoebocyte lysate, leading to the coagulation of the lysate. The GM test detects galactomannan (GM), a polysaccharide widely found in the cell walls of Aspergillus and Penicillium species. Galactomannan is released from the tips of fragile hyphae during hyphal growth and is one of the first antigens to be released. The G + GM test is mainly used clinically for the early diagnosis of invasive fungal infections, particularly invasive aspergillosis (6).

**Table 1:** List of mNGS results.

Sample name	Pathogen	Number of sequences
Bronchoalveolar lavage fluid	*Pseudomonas aeruginosa*	16,782
	*Enterococcus faecium*	15,703
	*Klebsiella pneumoniae*	16,280
	*Candida albicans*	17,703
	*Candida tropicalis*	17,301
	*Human betaherpesvirus 5*	17,945
Peripheral blood	*Aspergillus fumigatus*	12
	*Human polyomavirus 2*	2
Cerebrospinal fluid	*Aspergillus fumigatus*	434

## Discussion

With the aging population, increased incidence of degenerative heart valve disease, and more frequent use of prosthetic heart valves, the incidence and mortality of infective endocarditis have risen over the past 30 years (2). Streptococci and staphylococci are the most common pathogens (2). Fungal endocarditis, comprising 1%–3% of all cases, has a high mortality rate of 50%–95% (1, 3, 7). Among aspergillus the fumigatus type is the most common cause, followed by Aspergillus terreus, Aspergillus niger, and Aspergillus flavus (1, 8–10). Susceptible patients are typically male with underlying conditions such as cardiac abnormalities, prosthetic valves, malignancies, organ transplants, or immunosuppression (11–13). Native valve aspergillus endocarditis is rare (14). The incidence of invasive fungal endocarditis has increased significantly after coronavirus disease 2019 (COVID-19) (15). The patient had high-risk factors for Aspergillus endocarditis, including advanced age, prostate cancer, and recent COVID-19 infection. However, the atypical early echocardiographic findings led us to misjudge the condition as ischemic heart disease with heart failure, resulting in a delayed diagnosis.

Diagnosing Aspergillus endocarditis is challenging, with approximately 93% of cases reported as blood culture-negative (16). In such cases, 1,3-β-D-glucan (G) and galactomannan (GM) are often used but have limited diagnostic value due to low specificity and sensitivity (17). A small study found that serum G and GM tests had positivity rates of 85.7% and 62.5%, respectively, with over 20% of patients testing negative across all methods (7). Metagenomic next-generation sequencing has improved diagnostic accuracy and speed but is limited by high costs (18). Early diagnosis is crucial for improving prognosis, especially in patients with severe, rapidly progressing symptoms. In this case, mNGS identified the rare pathogen causing infective endocarditis, while blood and cerebrospinal fluid cultures, as well as serum G and GM tests, were repeatedly negative. The mitral valve mass was also discovered later through a follow-up echocardiogram.

mNGS (metagenomic Next-Generation Sequencing) is a high-throughput genomic technology used for comprehensive analysis of all microorganisms’ DNA or RNA in complex samples. Unlike traditional microbial culture or PCR methods, mNGS can simultaneously detect various microorganisms, including bacteria, viruses, fungi, and parasites, regardless of whether they are known or capable of growing on culture media. The main advantages of mNGS include the absence of the need to pre-select specific pathogens, its high sensitivity and broad spectrum, no culture requirement, and its automated high-throughput analysis (19–21). In the cardiovascular field, mNGS is used to assist in diagnosing infections with unclear etiology, such as endocarditis, by detecting pathogens that may be missed by conventional culture methods. This has significant implications for the early diagnosis and treatment of infectious heart diseases, especially in cases where antibiotic treatment fails or the cause remains unidentified. Despite its potential, mNGS has not yet become a routine diagnostic tool due to its high cost and the complex data analysis required, though it is gradually gaining wider adoption.

**Figure 4:**
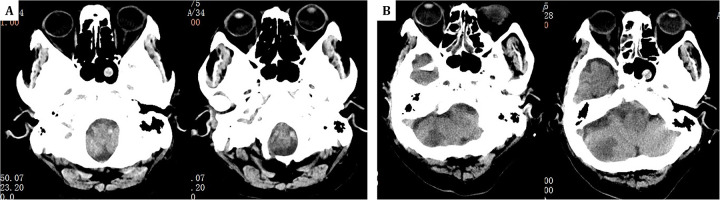
**(A)** One year prior, head CT revealed only a sphenoid sinus cyst. **(B)** The current admission's follow-up head CT at the same level now shows the sphenoid sinus cyst along with additional findings of right-sided ethmoid sinusitis.

In this case, mNGS quickly identified the pathogen and helped diagnose Aspergillus meningoencephalitis, suggesting a possible nasal sinus origin, as bronchoalveolar lavage did not detect *Aspergillus fumigatus* and newly developed sinusitis was noted on cranial CT (Figure 4). Common complications of Aspergillus endocarditis include cardiac issues, arterial embolism, mycotic aneurysms, metastatic abscesses, and neurological complications (22, 23). Embolization occurs in 54% of cases, most frequently affecting the brain, but also the spleen, kidneys, and lungs (1, 4). This patient experienced cerebral embolism and meningoencephalitis, with early recurrent focal neurological symptoms and imaging showing multiple cerebral emboli.

Aspergillus endocarditis has a poor prognosis and limited treatment options. Valvular vegetations often cause severe complications and high mortality. Conservative drug therapy is usually ineffective, and valve surgery is often required for embolic events (1, 5). Survival rates are 4% with antifungal therapy alone and 32% with surgery (24). In this case, delayed diagnosis led to cerebral hemorrhage. The need for valve surgery increased the risk of further bleeding, and the family opted for conservative treatment, which was ineffective. The patient ultimately developed bilateral pupil dilation, indicating a very poor prognosis.

## Conclusion

Aspergillus fumigatus endocarditis on native valves is rare but associated with high mortality. Effective therapeutic strategies are still lacking. Our experience suggests that for susceptible populations, such as older adults, immunocompromised individuals, and cancer patients, early diagnosis and treatment should be pursued promptly using mNGS when clinical symptoms, signs, and ultrasound findings strongly indicate the condition.

## Data Availability

The datasets presented in this article are not readily available because of ethical and privacy restrictions. Requests to access the datasets should be directed to the corresponding authors.
